# BOUNCING BACK AFTER SURGERY: QUANTIFYING AND PREDICTING RESILIENCE IN OLDER SURGICAL PATIENTS

**DOI:** 10.1093/geroni/igad104.0373

**Published:** 2023-12-21

**Authors:** Geeske Peeters, Heather Whitson

## Abstract

During this symposium we will discuss the impact of surgery on older adults physical functioning through a resilience lens. Physical resilience is defined as bouncing back in terms of physical functioning in response to a stressor, i.e. surgery. The first speaker will present data of long term trajectories of physical functioning in older women pre and post hip and knee surgery, and compare these with the trajectories in the general population. Profiles of women with higher or lower resilience to surgery will be discussed. The second speaker will talk about which functional measures, such as grip strength and walk speed, predict recovery in physical daily activities after knee replacement surgery, and the implications of these findings for the development of provocative resilience tests. The third presentation explores the role of biological ageing in fitness for surgery. The final presentation unravels the role of the stressor by exploring variations in types and severity of stressors in patients undergoing knee replacement surgery, and tests the hypothesis that severer stressors are associated with worse outcomes. Together, these presentations give a broad overview of determinants of physical resilience in the context of preparedness for, and recovery from, surgery. We will discuss methods to define stressors, and measure and quantify resilience. Moreover, we will illustrate how this information can inform personalization of care.

